# Community composition drives siderophore dynamics in multispecies bacterial communities

**DOI:** 10.1186/s12862-023-02152-8

**Published:** 2023-09-01

**Authors:** Siobhán O’Brien, Christopher T. Culbert, Timothy G. Barraclough

**Affiliations:** 1 Department of Microbiology, School of Genetics and Microbiology, Moyne Institute of Preventive Medicine, Trinity College Dublin, Dublin 2, Ireland; 2 Department of Life Sciences, Imperial College London, Silwood Park Campus, Ascot, Berkshire, SL5 7PY UK; 3 Department of Biology, University of Oxford, 11a Mansfield Road, Oxford, OX1 3SZ UK

**Keywords:** Microbial communities, Public goods, Species interactions, Community ecology

## Abstract

**Background:**

Intraspecific public goods are commonly shared within microbial populations, where the benefits of public goods are largely limited to closely related conspecifics. One example is the production of iron-scavenging siderophores that deliver iron to cells via specific cell envelope receptor and transport systems. Intraspecific social exploitation of siderophore producers is common, since non-producers avoid the costs of production but retain the cell envelope machinery for siderophore uptake. However, little is known about how interactions between species (i.e., interspecific interactions) can shape intraspecific public goods exploitation. Here, we predicted that strong competition for iron between species in diverse communities will increase costs of siderophore cooperation, and hence drive intraspecific exploitation. We examined how increasing microbial community species diversity shapes intraspecific social dynamics by monitoring the growth of siderophore producers and non-producers of the plant-growth promoting bacterium *Pseudomonas fluorescens*, embedded within tree-hole microbial communities ranging from 2 to 15 species.

**Results:**

We find, contrary to our prediction, that siderophore production is favoured at higher levels of community species richness, driven by increased likelihood of encountering key species that reduce the growth of siderophore non-producing (but not producing) strains of *P. fluorescens*.

**Conclusions:**

Our results suggest that maintaining a diverse soil microbiota could partly contribute to the maintenance of siderophore production in natural communities.

**Supplementary Information:**

The online version contains supplementary material available at 10.1186/s12862-023-02152-8.

## Introduction

Microbes exhibit a wide range of cooperative behaviours that can shape, and be shaped by, the communities within which they reside [1, 2]. Siderophore production by bacteria and fungi is one well-studied example of a cooperative behaviour [3–5]. Under iron-limitation, extracellular siderophores are produced that deliver iron to the cell via specific receptor and transport systems [6]. However, since siderophore production is metabolically costly, it can be exploited by non-producing cheats who avoid the cost of production while benefiting from siderophores of nearby cooperators [3]. Such non-producers invade populations of cooperators under iron-limited conditions and can evolve *de novo* within days [7, 8].

Selection for non-producing phenotypes depends on the relative costs and benefits of siderophore production. The cost of siderophore production increases as iron becomes limited and genes associated with siderophore production are upregulated [9]. When costs of cooperating are higher, there is increased selection for cheating, as non-producers experience a large relative fitness advantage by avoiding or reducing the cost of cooperating. Paradoxically, this means that cheating is strongly favoured when siderophores carry large benefits [9, 10]. Cooperators, on the other hand, will benefit from siderophore production when these benefits are more likely to accrue to the cooperators themselves or identical clonemates who also produce siderophores, for example via spatial heterogeneity or environmental viscosity [11, 12].

While the role of intraspecific interactions is well-established in driving siderophore dynamics, less is known about how interspecific interactions alter the dynamics of within-species siderophore cooperation in microbial communities. Given that species can compete with one another for iron in natural ecosystems [13, 14], provide a source of iron upon cell lysis [15], or even pirate ‘foreign’ siderophores produced by other species [16], it is likely that species interactions can alter the costs and benefits of siderophore production in multiple, contrasting ways. For example, the presence of a second species (*Staphylococcus aureus*) can either promote or restrict the invasion of *Pseudomonas aeruginosa* siderophore non-producing cheats by acting as a competitor or source of iron, respectively - depending on the degree of competition between the two species [15, 17]. However, our understanding of how species interactions alter intraspecific siderophore production dynamics in natural communities remains largely limited to the aforementioned highly simplified two-species communities. In natural complex communities, a high degree of competition for resources such as iron [13, 14] or even the building blocks of siderophores themselves [18], could increase selection for non-producing cheats by increasing the cost of cooperation. This is important, as siderophore-producing bacteria are often relied upon for functions such as plant growth promotion and biocontrol, where they must interact with diverse soil, root or leaf microbiomes [19].

Here, we test whether increasing microbial community species diversity shapes selection for non-producers, by monitoring the growth of siderophore producers and non-producers of the plant-growth promoting bacterium *Pseudomonas fluorescens*, embedded within communities ranging from 2 to 15 species. We predicted that siderophore exploitation should be greatest in high diversity communities, where between-species competition is more intense. Community taxa originated from semi-permanent rain-filled wells formed by the roots of beech trees (*Fagus sylvatica*) [20], and competition has previously been reported to dominate between wild isolates of these taxa [21]. We find, contrary to our prediction, that siderophore production is favoured in higher (versus low) diversity communities, suggesting that maintaining a healthy soil microbiota could contribute to the maintenance of siderophore production in natural communities.

## Methods

### Focal species– ***Pseudomonas fluorescens***

We used the gentamicin-resistant *lac-Z* marked *Pseudomonas fluorescens* strain SBW25-*lacZ* as our siderophore producing strain [22] and the gentamicin-resistant strain SBW25Δ*pvdL* which lacked genes encoding the primary siderophore pyoverdine, as our non-producing strain [23]. Gentamicin resistance distinguished our focal strains from the rest of the community (see below). *LacZ* conferred producers (SBW25-*lacZ*) a blue pigment, so that they could be easily distinguished from non-producers (SBW25Δ*pvdL*) on Lysogeny Broth (LB) agar supplemented with 90 μg/mL 5-Bromo‐4‐chloro‐3‐indolyl‐β‐Dgalactopyranoside (X‐gal). Previous work has shown that fitness levels of SBW25-*lacZ* are comparable to the wild-type ancestor, suggesting the cost of the *lacZ* marker is negligible or absent [22].

### Background microbial community

We screened 230 isolates from an archived tree-hole library [24] for (i) gentamicin susceptibility (to permit differentiation from focal *P. fluorescens* strains) and (ii) growth in iron-limited lysogeny broth (LB) (to ensure survival under the experimental conditions). Gentamicin susceptibility of each isolate was tested using 10 μg gentamicin antimicrobial susceptibility discs (Thermo Scientific™ Oxoid™). Growth in iron-limited LB was verified by measuring OD600 (600 nm) of each isolate after 48 h at 22^O^C in iron-limited LB (LB supplemented with 100 μg/mL human apo-transferrin, an iron chelator, and 20 mM sodium bicarbonate [25]). This screening process produced a library of fifteen isolates from which we built our background communities. The identities of the chosen 15 isolates (Table 1) were confirmed through 16 S rRNA sequencing (supplementary methods).

**Table 1:** Bacterial genera used in this study

Strain	Genus	Experimental number
BB19.36	*Yersinia*	**1**
SP01.03	*Acinetobacter*	**2**
SP01.04	*Bacillus*	**3**
SP03.13	*Janthinobacter*	**4**
SP06.03	*Erwinia*	**5**
SP03.19	*Epilithonimonas*	**6**
WYM27.02	*Pantoea*	**7**
WYM29.03	*Rhodococcus*	**8**
WYC41.02	*Pseudomonas*	**9**
BB66.01	*Aeromonas*	**10**
SP03.21	*Serratia*	**11**
SP04.06	*Pedobacter*	**12**
AE95.04	*Stenotrophomonas*	**13**
BB19.34	*Chryseobacter*	**14**
SP06.07	*Buttiauxella*	**15**

### Experimental design

We tested whether the growth of our focal *P. fluorescens* producer and non-producer was affected by the species richness of the background community. We chose four levels of community richness: 2, 4, 8, and 15. Each richness level includes the addition of *P. fluorescens*, for example, a community richness of 4 represents *P. fluorescens* plus 3 other species. We adopted a random sampling design (see [26]). Each richness level was represented by 5 random combinations of the 15 bacterial isolates, except when richness was equal to two; when equal to two, all 15 background isolates were grown with SBW25. Therefore, the experiment included 15 different two-species communities, 5 different four-species communities, 5 different eight-species communities and 5 different fifteen-species communities (Supplementary Table S1). Each community was replicated five times for both single and mixed *P. fluorescens* treatments (see below).

To test whether our focal *P. fluorescens* non-producer could exploit siderophores of the *P. fluorescens* producer under different levels of community diversity, we grew our focal *P. fluorescens* strains as single genotypes (producer and non-producer grown separately) and as mixed genotypes (producer and non-producer grown together in a 1:1 mixture). Including a mixed versus single genotype treatment allowed us to confirm that between-species siderophore exploitation could occur in this particular environment. Initial densities of our focal *P. fluorescens* species were always the same between single and mixed genotype conditions (i.e. as mixed genotypes, the starting densities of each strain was halved compared to single genotypes). Individual communities were assembled using a liquid handling robot (Hamilton Microlab STARlet), so all taxa within a community were inoculated at equal starting densities. The starting density for each community was always ~ 400 cells/well (enumerated by the flow cytometry of stock solutions). For example, in 4-species communities, ~ 100 cells of *P. fluorescens* were inoculated alongside ~ 100 cells each of 3 different species. 20 μl of the assembled community was added to 180 μl iron-limited LB media in 96 well microplates (Thermo Fisher Scientific) to a final volume of 200 μl. Plates were incubated statically, at room temperature for 7 days as batch culture, to facilitate growth of both our focal strain and slow-growing members of the community. Final cell densities of focal *P. fluorescens* were measured by plating on gentamicin-supplemented LB agar and counting colony forming units (CFU / ML).

### Statistical analysis

We first tested for evidence of exploitation within *P. fluorescens*, by comparing the final proportion of *P. fluorescens* non-producers in single versus mixed genotype communities. In single genotype treatments, we randomly paired a producer with a non-producer population from the same richness level, to calculate the proportion of non-producers. We used generalised mixed effects models (glmer) with a binomial error structure and community ID as a random factor, assigning species richness (factor) and growth condition (single versus mixed genotype) as explanatory variables, and proportion of non-producers as a response variable. We next estimated the total final population density (CFU / ML) for each *P. fluorescens* strain (producer or non-producer) in each community under both single and mixed genotype conditions. For single genotype conditions, we tested whether species richness (fitted as a factor) and/or *P. fluorescens* genotype (producer or non-producer) affected final densities of *P. fluorescens*, using lmer, controlling for the identity of the background community as a random factor. The response variable (final densities) was log transformed to comply with model assumptions of normality. For mixed genotype conditions, we tested whether the proportion of non-producers in each community was affected by species richness (fitted as a factor) in a glmer with a binomial error structure and community as a random factor. Lastly, we quantified the effect of each species on final densities of producers and non-producers as single genotypes, by using two separate linear models to partition the variance between community richness and species ID in explaining siderophore producer final cell densities. The species coefficients provided by this method give a measure of the effect of each species on focal strain final densities relative to an average species. We analysed data using R version 4.1.2.

## Results

### Evidence for exploitation within the focal species

By comparing the final proportion of *P. fluorescens* non-producers in single versus mixed genotype communities, we find that mean proportion of non-producers increased when in co-culture with the producer (glmer; effect of mixed/single genotype, *X*^2^_1,5_= 36.19, p < 0.0001). This was irrespective of species richness level (glmer; mixed/single genotype x richness, *X*^2^_3,6_= 1.77, p = 0.62, Fig S1). In other words, non-producers reached higher frequencies in communities when their conspecific producer was also present – suggesting that within-species siderophore exploitation occurs in this system.

### ***P. fluorescens*** siderophore producer and non-producer growth increases and decreases respectively as communities become more diverse

#### Single genotype condition

**Fig. 1:**
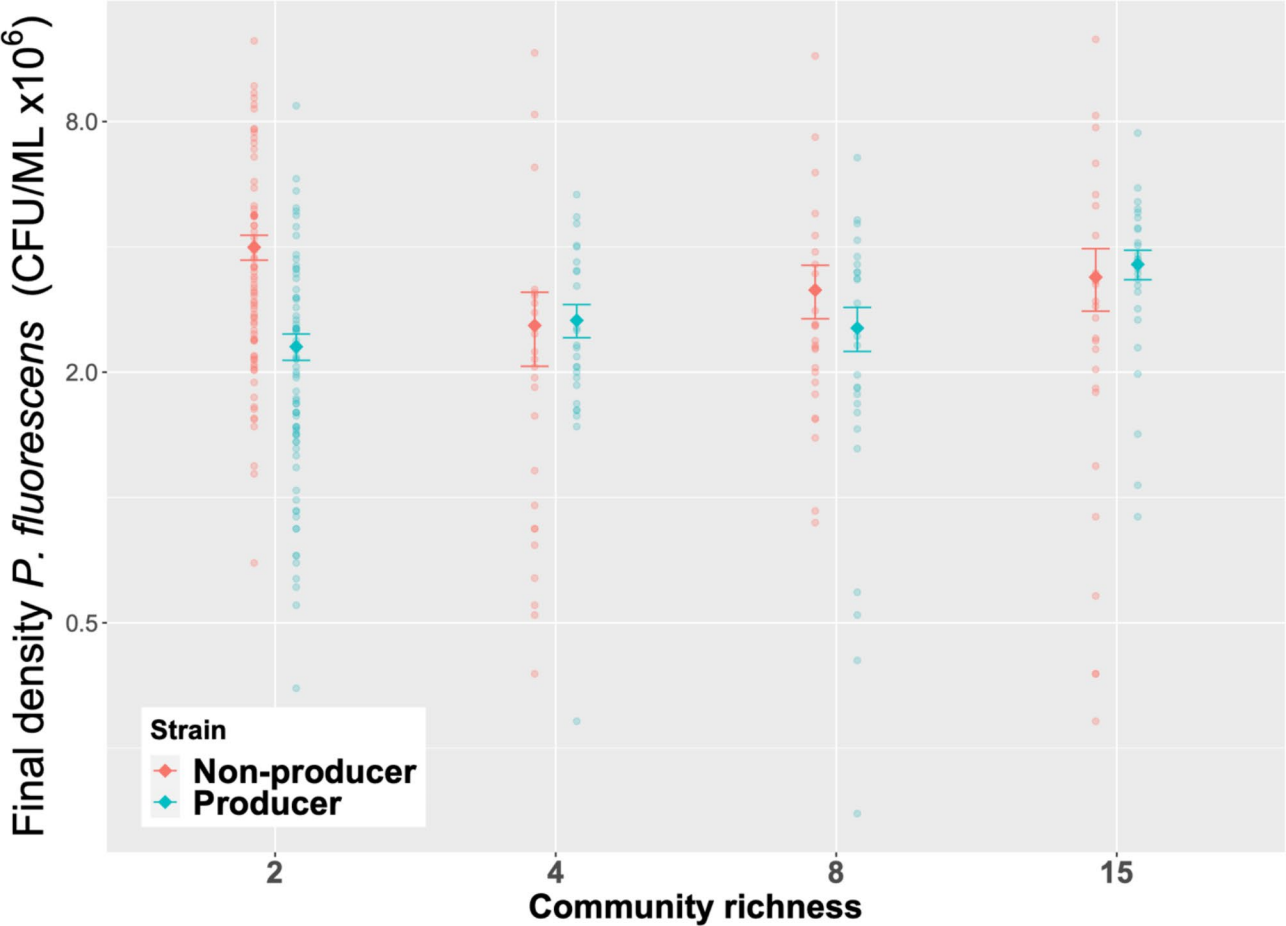
Final densities (log(CFU/ML x10^6^)) of single genotype *P. fluorescens* producer and non-producer populations in communities comprising 2, 4, 8 and 15 species. Final densities of producers did not differ significantly between richness levels, whereas final densities of non-producers decreased between richness levels two and four. See main text for statistics. Sample size varies between richness levels. When r = 2, n = 75. When r = 4,8 or 15, n = 25. Solid points show mean values ± SE. A full list of post-hoc results are available in Table S4

Under single genotype conditions (where the producer and non-producer are grown separately) species richness affected final densities of our focal producer and non-producer differently (lmer; genotype x richness; *X*^*2*^_3,7_^=^ 27.79, p < 0.0001, Fig. 1). Specifically, final densities of producers did not differ significantly between richness levels (TukeyHSD, p > 0.05 in all cases). However, for non-producers, we find a significant reduction in final densities between richness levels two and four (TukeyHSD, p < 0.0001). There were no obvious differences in non-producer densities between the remaining richness levels (TukeyHSD, p > 0.05 in all cases).

We next compared final densities of producer versus non-producers within the same richness level. We find that while non-producers reach significantly greater (173%) final densities relative to producers in two-species communities (TukeyHSD p < 0.001, Fig. 1), this advantage is lost in communities comprising 4, 8 and 15 species, where there was no difference between producer and non-producer final cell densities (TukeyHSD p > 0.05 in all cases, Fig. 1).

#### Mixed genotype condition

Under mixed genotype conditions (where the producer and non-producer are grown together), we hypothesised that increased competition for iron in species rich communities should increase the growth of non-producers relative to conspecific producers. This is because the cost of producing siderophores increases as iron becomes more limited, and non-producers can avoid these costs by exploiting siderophores of their conspecific producer [9].

**Fig. 2:**
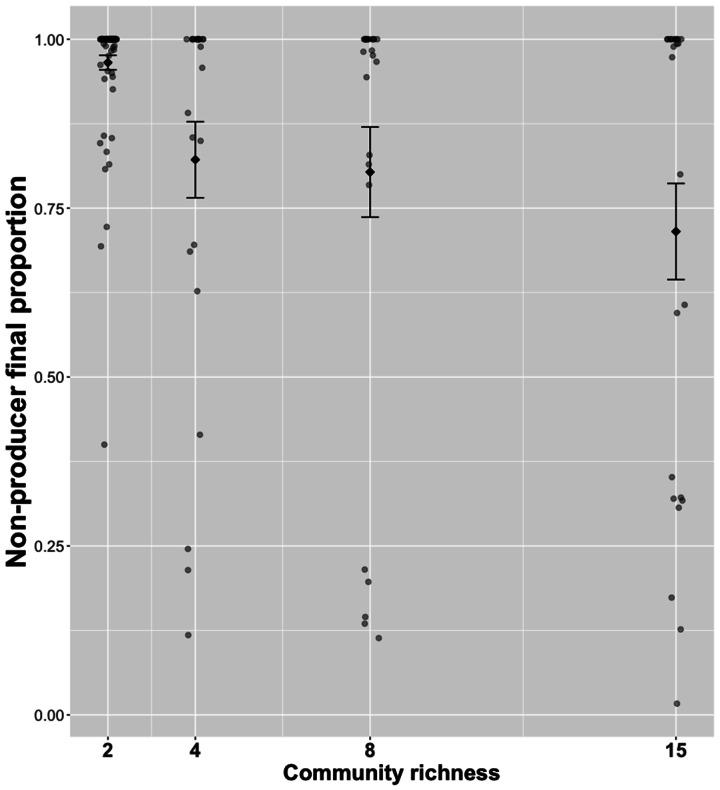
Non-producer final proportion in mixed genotype treatments at increasing richness levels. We find that the proportion of non-producers in the community reduced as species richness increased (see main text for statistics). Sample size varies between richness levels. When r = 2, n = 75. When r = 4,8 or 15, n = 25. Black diamonds show mean value ± SE. A full list of post-hoc results are available in Table S5

Contrary to our prediction, we found that when both producer and non-producer genotypes were grown together (mixed genotype treatment), the proportion of non-producers in the community decreased with increasing species richness (glmer; effect of richness; X^2^_3,2_= 12.86, p < 0.005, Fig. 2). The greatest reduction in the proportion of non-producers occurred between 2- and 15- species communities (Tukey HSD, p < 0.05). This is in line with our single genotype result (above) indicating that non-producer final densities reduced as communities became more diverse. Together, these findings show that while increased community complexity can contribute to the maintenance of siderophore production, this was independent of intraspecific exploitation (which could not occur in the single genotype treatment where non-producers only were added).

### Disentangling the effect of species richness versus composition on ***P. fluorescens*** producer and non-producer final cell densities

The above analyses revealed opposing effects of species richness on *P. fluorescens* producer and non-producer populations. However, the above models could not differentiate between the effect of richness per se or the added chance of including a biologically important species at high richness levels. Our replication of different compositions at each species richness level allowed us to further investigate the relative roles of richness versus composition on final cell densities [26].

We first investigated whether any particular species had stronger effects than average on final cell densities of our focal *P. fluorescens* producer and non-producer strains growing as single genotypes. In producer populations, species richness and species identity explained 10% and 18% of the variance in final population densities, respectively (Table S2). In non-producer populations, species richness and identity explained 4% and 11% of the variance in final population densities, respectively (Table S3). Together, this suggests that community species composition (rather than species richness per se) is the dominant driver of siderophore dynamics in our focal species.

**Fig. 3:**
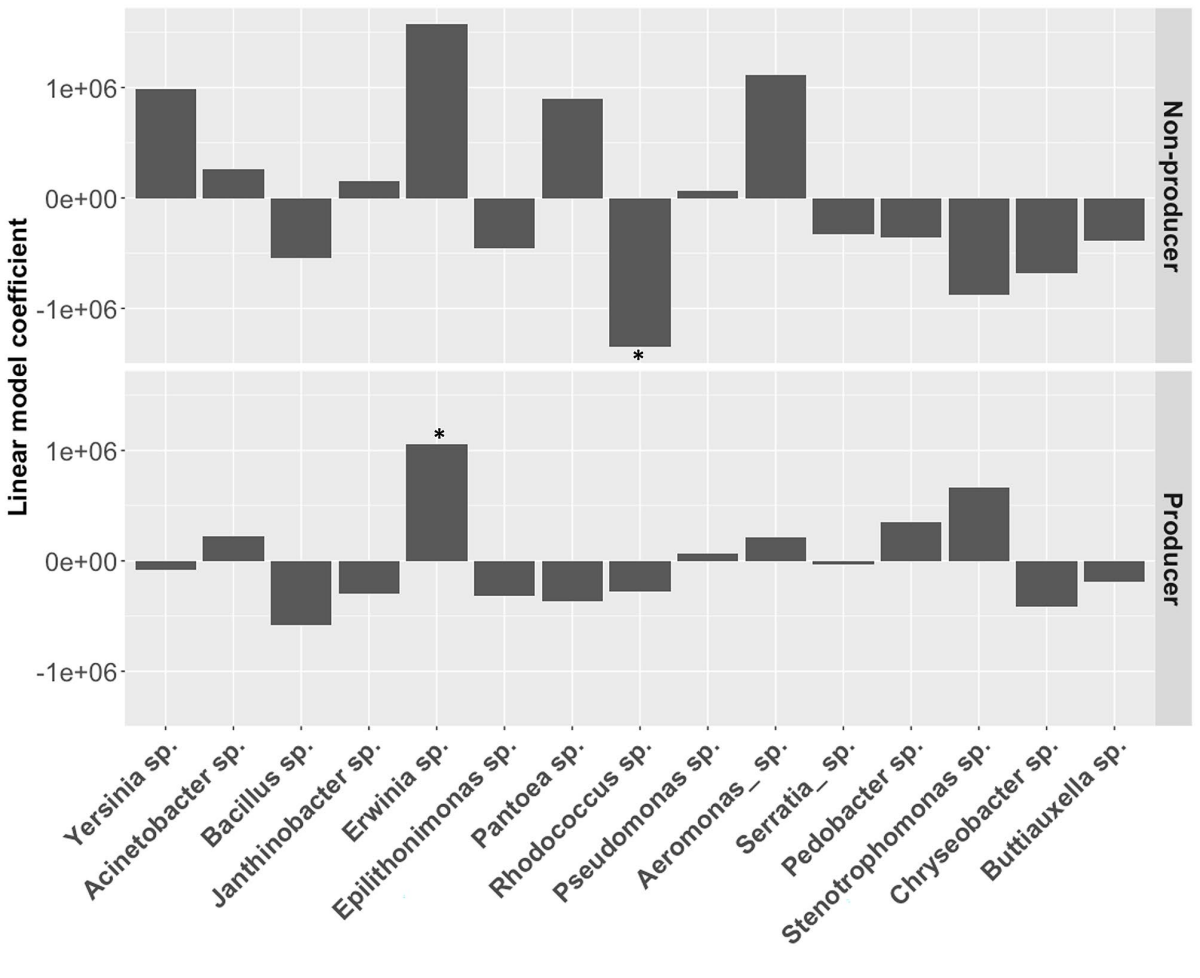
Linear model coefficients for *P. fluorescens* siderophore non-producers (top panel) and producers (bottom panel) strains grown as single genotypes. Positive or negative coefficients indicate species that contribute more or less to *P. fluorescens* growth compared to an average species [26]. We find that *Erwina sp* (sp. 5) significantly increases final densities of siderophore-producing *P. fluorescens* relative to other community species, while *Rhodococcus sp.* (sp. 8) reduces final densities of non-producers, relative to the effect of all other community species (see main text for statistics)

Our random sampling design allowed us to next identify key community species that either promoted or constrained the growth of our focal strain. Linear model coefficients provide the estimated contribution of each species to *P. fluorescens* final densities, relative to an average species’ contribution [26]. We identified one species, sp. 5 (*Erwina sp.)*, that was associated with higher *P. fluorescens* producer final densities relative to an average community species (producer: t_16,134_=2.19, p < 0.05, Fig. 3). *Erwina sp.* was also associated with higher final densities of non-producers, however, this effect was not statistically significant (t_16,134_=1.79, p = 0.07, Fig. 3*).* For non-producers, one species - *Rhodococcus sp* (sp8)- was associated with lower densities of non-producers (t_16,134_ = 2.01, p < 0.05, Fig. 3), yet had no detectible effect on producers (t_16,134_ = 0.761, p = 0.45, Fig. 3). Together, these findings suggest that the increased likelihood of encountering *Rhodococcus sp* at high richness levels can explain the apparent reduced cost of siderophore producers in diverse versus simple communities.

## Discussion

Here, we empirically test whether the costs and benefits of siderophore production in *P. fluorescens* depend on the composition and richness of the background microbial community. We hypothesised that increased competition for iron in species-rich communities should increase costs of cooperation and consequently increase selection for non-producers [13, 27]. However, we find the opposite - growth of non-producers is instead reduced in species-rich communities because there is an increased likelihood of encountering species that selectively reduce densities of non-producers when diversity is high. Our findings suggest that in natural microbial communities, maintaining a diverse microbiota may contribute to the maintenance of siderophore cooperation.

Our finding that siderophore exploitation did not favour non-producers in more diverse communities was surprising, since increased competition for resources in diverse communities are predicted to increase costs of cooperation and hence benefits of cheating [17]. In addition to our finding that non-producer-suppressing species could promote siderophore production, other ecological conditions could also have contributed to this effect. Firstly, cell counts were quantified after 7 days growth in batch culture. Siderophore producers are less exploitable in stationary phase [28] (since production itself is downregulated in late exponential and stationary growth phases), and species within more diverse communities tend to reach stationary phase earlier [29]. This implies that opportunities for siderophore exploitation (i.e. during mid-exponential phase) are reduced in species rich communities. Secondly, initial cell densities of *P. fluorescens* are higher in low diversity communities due to the nature of our design (i.e. starting total cell densities were equal for all communities). Within-species exploitation is greater at high cell densities, since cheats are better able to exploit producers when they are physically closer to them [30]. Hence, our low species richness communities (which were initiated with higher densities of *P. fluorescens* compared to high richness communities), potentially created more opportunities for siderophore exploitation compared to species rich communities. Ultimately, cooperation in communities is likely to be driven by complex interactions between biotic and abiotic factors, where the ecological effects of a community on a species’ cell densities and growth rates are experienced alongside the effects of species interactions themselves.

In 2-species communities, we find that non-producers reach higher final densities than producers when grown as single genotypes – where non-producers could not access conspecific siderophores. While this is a surprising result, we pose some possible explanations. Firstly, while the benefits of iron-chelating siderophores tend to be species-specific (due to the requirement of a species specific siderophore receptor), there has been some evidence of siderophore piracy occurring between species [31, 32]. Hence, non-producers may be able to exploit siderophores produced by the second species. Indeed, the ability to sequester unavailable iron (likely via siderophore production) was a key prerequisite for a community species to be included in our experiment (see methods). Furthermore, many of our community species are well-known to produce siderophores, such as Acinetobactor sp [33]., Bacillus sp [34]., Pantoea sp [35]., Serratia sp [36]., Pseudomonas sp [37]. and Aeromonas sp. (Note that the Pseudomonas isolate in our background community displayed a closest 16 S-rDNA match to P. *poae*, which is in the *P. fluorescens* sub-group of the genus, but relatively divergent from our focal species *P. fluorescens* SBW25 [38]. Secondly, there is some evidence that dead cells can be used as a source of iron for growing populations [15]. Since our final cell counts were taken after 7 days (i.e. when populations were in late stationary phase), cell death could have played a significant role in allowing non-producers to access iron. Finally, while our focal strains differed in their ability to produce the primary high-affinity siderophore pyoverdine [23], both strains retain the ability to produce secondary siderophores (e.g. ornicorrugatin in SBW25 [39]), so total SBW25ΔpvdL siderophore production is ~ 21% of the SWBW5 wildtype [40]. Secondary siderophores are expressed under moderate iron-limitation, where the metabolic costs of producing pyoverdine may not outweigh the benefits [41]. Furthermore, there is some evidence that pyoverdine can repress the production of secondary siderophores [41], potentially explaining why non-producers grow as well as, or better than, producers under low levels of community richness. Extending this further, we could speculate that increased competition for iron as community diversity increases favours a switch to pyoverdine production in species-rich communities. Our results suggest a key avenue for further research is the interplay between siderophore regulation and competition for iron in natural microbiota.

Our experiments support the idea that genotypic variation within a focal strain (in this case, a single SNP in *pvdL*) can markedly shape the nature of interactions between species in a community [42]. Species interactions in a community can in turn alter the outcome of competition between genotypes within a species – especially when interacting species have genotypic-specific effects [42]. Our study identified a key species in the community (*Rhodococcus sp)* that could alter the dynamics of public goods production in our focal species. It is unclear how siderophore-production could drive the outcome of interactions between *P. fluorescens* and *Rhodococcus sp.* However, intraspecific variation in *Pseudomonas aeruginosa* virulence-associated secretions have been found to drive the outcome of *P. aeruginosa* – *Staphylococcus aureus* interactions from competition to coexistence in chronic respiratory infections [43]. Hence, interspecific interactions in communities can have important consequences for within-species dynamics. We had no prior hypotheses for which species should particularly influence the relative performance of producers and non-producers, and future work would be needed to determine why *Rhodococcus* sp. in particular had strain-specific effects on *P. fluorescens.* We note that our random sampling design was not a true random partition design described in [26], since logistical constraints meant that we were not able to repeat the species selection process at each richness levels. Hence, it is possible that by chance, some species are over-represented at each richness level. However, we can speculate that *Rhodococcus*, a siderophore producing genus [44], might outcompete *P. fluorescens* non-producers for iron using siderophores that are not accessible for uptake by *P. fluorescens* non-producers.

Finally, although we are limited here to just 15 species and one community type (tree-hole) our results pose interesting questions about the role of the microbiota in maintaining public goods production. Siderophore production is key for *P. fluorescens* as a biocontrol agent against plant pathogens [19] - yet siderophore non-producing genotypes abound in nature that can compromise its efficacy. It is possible that maintaining a healthy diverse soil microbiota could constrain the spread of non-producing genotypes. More generally, the production of public goods such as siderophores, antibiotic resistance via excreted detoxifying systems, and secreted virulence factors have important implications for patient health, ecosystem services and biotechnology. It is therefore important to understand how these systems are affected by the presence of additional non-focal taxa in naturally diverse microbiomes.

### Electronic supplementary material

Below is the link to the electronic supplementary material.

Supplementary Material 1: Methods

Supplementary Material 2: Figures 

Supplementary Material 3: Tables

Supplementary Material 4: Dataset

## Data Availability

All data generated or analysed during this study are included as electronic supplementary material.
