# Supplementary material for: Community composition drives siderophore dynamics in multispecies bacterial communities
Source: BMC Ecol Evol. 2023 Sep 1;23:45. doi: 10.1186/s12862-023-02152-8 (PMC10472669; doi:10.1186/s12862-023-02152-8)
Supplement: Supplementary file 1 — Supplementary Material 1: Methods [file 12862_2023_2152_MOESM1_ESM.docx]

**Supplementary Methods**

**16S sequencing**: DNA was extracted from the twelve isolates using a sodium dodecyl sulphate (SDS) extraction method: A loop of bacteria from culture was suspended in 0.5% SDS; the isopropanol-precipitated DNA was pelleted by spinning at 13,100 rpm for 10 min in a centrifuge, and the pellet was washed in ethanol and re-suspended in 50 μl of sterile water. All chemicals used were obtained from VWR International, except proteinase K and cetrimonium bromide (Sigma-Aldrich). PCR-amplification of 1,500 bp fragments of 16S rDNA was performed using oligonucleotide primers 27F (5’-AGAGTTTGATCMTGGCTCAG- 3’) and 1492R (5’-TACGGYTACCTTGTTACGACTT-3’) (InvitrogenTM, Thermo Fisher Scientific), using the Mastercycler® gradient thermo-cycler (Eppendorf). The PCR reaction mix of 25μl per sample contained 2μl of template DNA, 12.5μl of RedTaq®, 1.5μl of each primer and 7.5μl of molecular-grade water (Sigma-Aldrich). Reaction mixes were loaded into the thermo-cycler to amplify fragments. PCR products were verified using gel electrophoresis. The 16S rDNA amplicons from 12 isolates were sent to Macrogen© for Sanger sequencing (Sanger et al., 1977). Forward and reverse sequences were aligned using the bioinformatics software Geneious© (Biomatters) and the consensus sequences were manually cleaned in a conservative manner to ensure accuracy. The consensus sequences were then used to identify the isolates via BLAST (Altschul et al., 1990) and using the Sequence Match function in Ribosomal Database Project (Cole et al., 2014).

Altschul, S.F., Gish, W., Miller, W., Myers, E.W., Lipman, D.J. (1990) Basic local alignment search tool. Journal of molecular biology. 215(3), 403–10.

Cole, J.R., Wang, Q., Fish, J. a, Chai, B., McGarrell, D.M., Sun, Y., Brown, C.T., Porras- Alfaro, A., Kuske, C.R., Tiedje, J.M. (2014) Ribosomal Database Project: data and tools for high throughput rRNA analysis. Nucleic acids research. 42(Database issue), D633–42.
