# Supplementary material for: Community composition drives siderophore dynamics in multispecies bacterial communities
Source: BMC Ecol Evol. 2023 Sep 1;23:45. doi: 10.1186/s12862-023-02152-8 (PMC10472669; doi:10.1186/s12862-023-02152-8)
Supplement: Supplementary file 2 — Supplementary Material 2: Figures [file 12862_2023_2152_MOESM2_ESM.docx]

**Supplementary Figure S1.** Final proportion of non-producers to producers of *P. fluorescens* comparing treatments where they grew together in a mixture (mixed genotype treatment) or separately (single genotype treatment). Mean and standard errors for different species richness levels are shown. Non-producers reached higher frequencies when inoculated in the presence of a producer (i.e. mixed genotype treatments). This effect was independent of community richness. See main text for statistics.


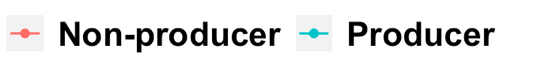


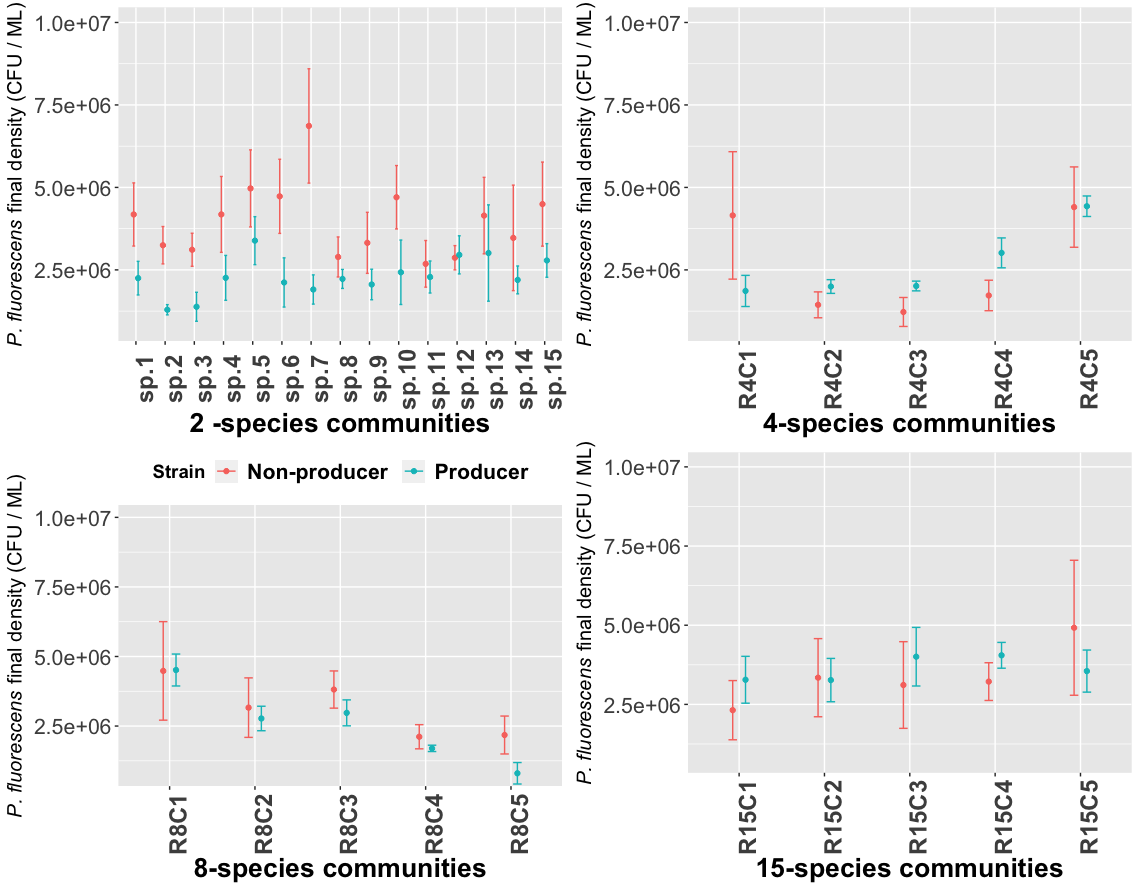


**Supplementary Figure S2.** *P. fluorescens* final densities (CFU/ ML) after growing individually as single genotypes, in communities ranging from 2-15 species. We find significantly greater (173%) non-producer (orange) versus producer (blue) final densities in two-species communities, but no difference between producer and non-producer final cell densities in 4, 8 and 15 species communities (see main text for statistics). Mean and standard errors for different species richness levels are shown.
