# Supplementary material for: Community composition drives siderophore dynamics in multispecies bacterial communities
Source: BMC Ecol Evol. 2023 Sep 1;23:45. doi: 10.1186/s12862-023-02152-8 (PMC10472669; doi:10.1186/s12862-023-02152-8)
Supplement: Supplementary file 3 — Supplementary Material 3: Tables [file 12862_2023_2152_MOESM3_ESM.docx]

| Richness | Community ID  ID ID | Species included |
| --- | --- | --- |
| 2 | Sp.1 | 1 |
| 2 | Sp.2 | 2 |
| 2 | Sp.3 | 3 |
| 2 | Sp.4 | 4 |
| 2 | Sp.5 | 5 |
| 2 | Sp.6 | 6 |
| 2 | Sp.7 | 7 |
| 2 | Sp.8 | 8 |
| 2 | Sp.9 | 9 |
| 2 | Sp.10 | 10 |
| 2 | Sp.11 | 11 |
| 2 | Sp.12 | 12 |
| 2 | Sp.13 | 13 |
| 2 | Sp.14 | 14 |
| 2 | Sp.15 | 15 |
| 4 | R4C1 | 1, 4, 11 |
| 4 | R4C2 | 3, 6, 9 |
| 4 | R4C3 | 6, 7, 8 |
| 4 | R4C4 | 4, 8, 9 |
| 4 | R4C5 | 2, 10, 12 |
| 8 | R8C1 | 2, 5, 7, 9, 11, 13, 15 |
| 8 | R8C2 | 1, 3, 5, 8, 9, 12,14 |
| 8 | R8C3 | 2, 3, 4, 7, 10, 11, 13 |
| 8 | R8C4 | 1, 4, 6, 7, 13, 14, 15 |
| 8 | R8C5 | 3, 4, 7, 8, 10, 12, 15 |
| 15 | R15C1 | 2, 3, 4, 5, 6, 7, 8, 9, 10, 11, 12, 13, 14, 15 |
| 15 | R15C2 | 1, 2, 4, 5, 6, 7, 8, 9, 10, 11, 12, 13, 14, 15 |
| 15 | R15C3 | 1, 2, 3, 4, 5, 6, 8, 9, 10, 11, 12, 13, 14, 15 |
| 15 | R15C4 | 1, 2, 3, 4, 5, 6, 7, 8, 10, 11, 12, 13, 14, 15 |
| 15 | R15C5 | 1, 2, 3, 4, 5, 6, 7, 8, 9, 10, 11, 12, 14, 15 |

**Table S1:** Using a random sampling experimental design (see [26]), we tested whether the growth of our focal *P. fluorescens* producer and non-producer was affected by the richness of the background community. Four levels of community richness: 2, 4 ,8, and 15 was tested. Each richness level includes the addition of *P. fluorescens*, for example, a community richness of 4 represents *P. fluorescens* plus 3 other species. Each richness level was represented by 5 random combinations of the 15 bacterial isolates, except when richness was equal to two; when equal to two, all 15 background isolates were grown with *P. fluorescens*. Each community was replicated five times for both single and mixed genotype conditions.

**Table S2:** ANOVA summary table for linear models partitioning the variance between species ID and community richness in explaining siderophore producer final cell densities. Values were generated in R using the tab_model function (sjPlot package).


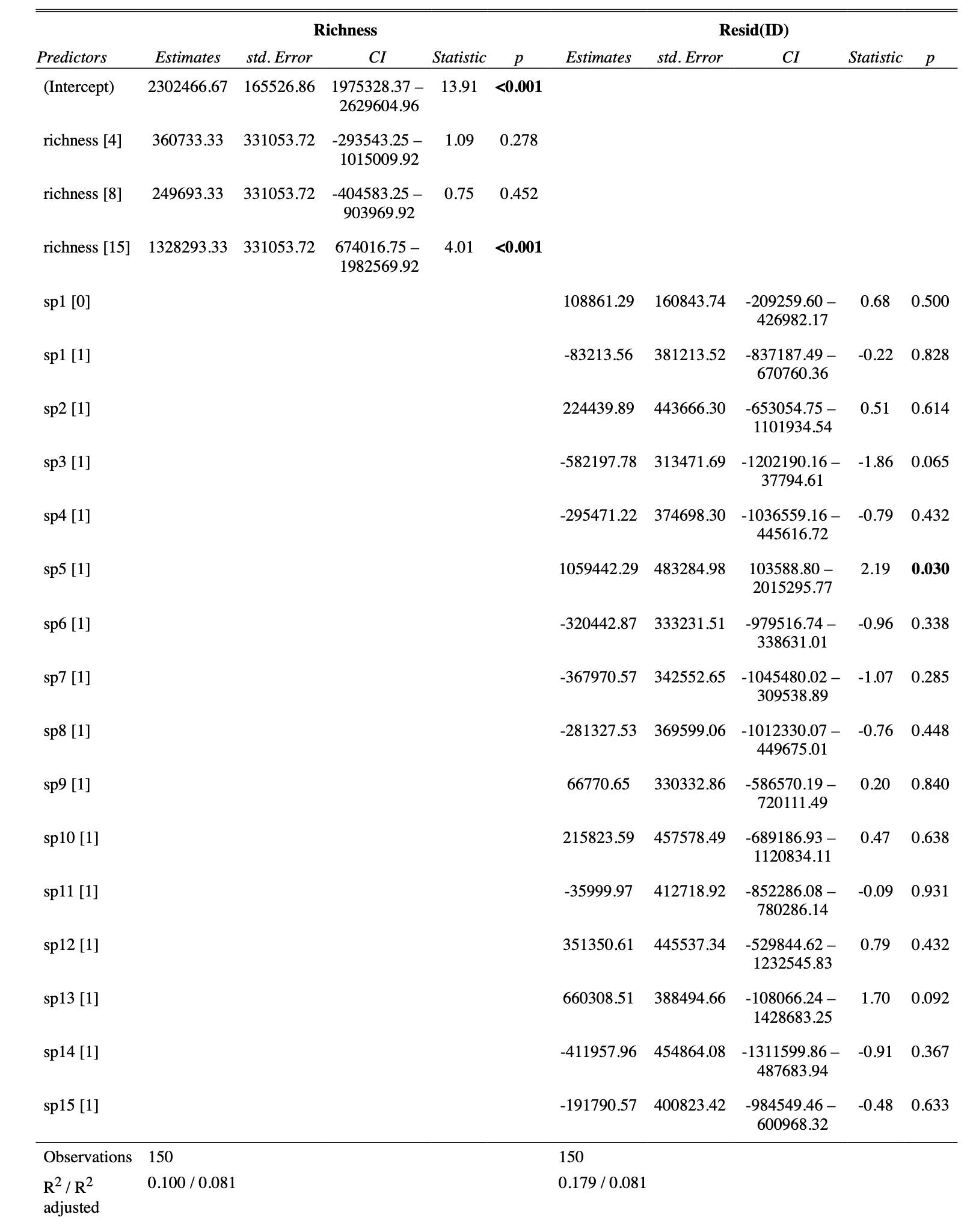


**Table S3:** ANOVA summary table for linear models partitioning the variance between species ID and community richness in explaining siderophore non-producer final cell densities. Values were generated in R using the tab_model function (sjPlot package).


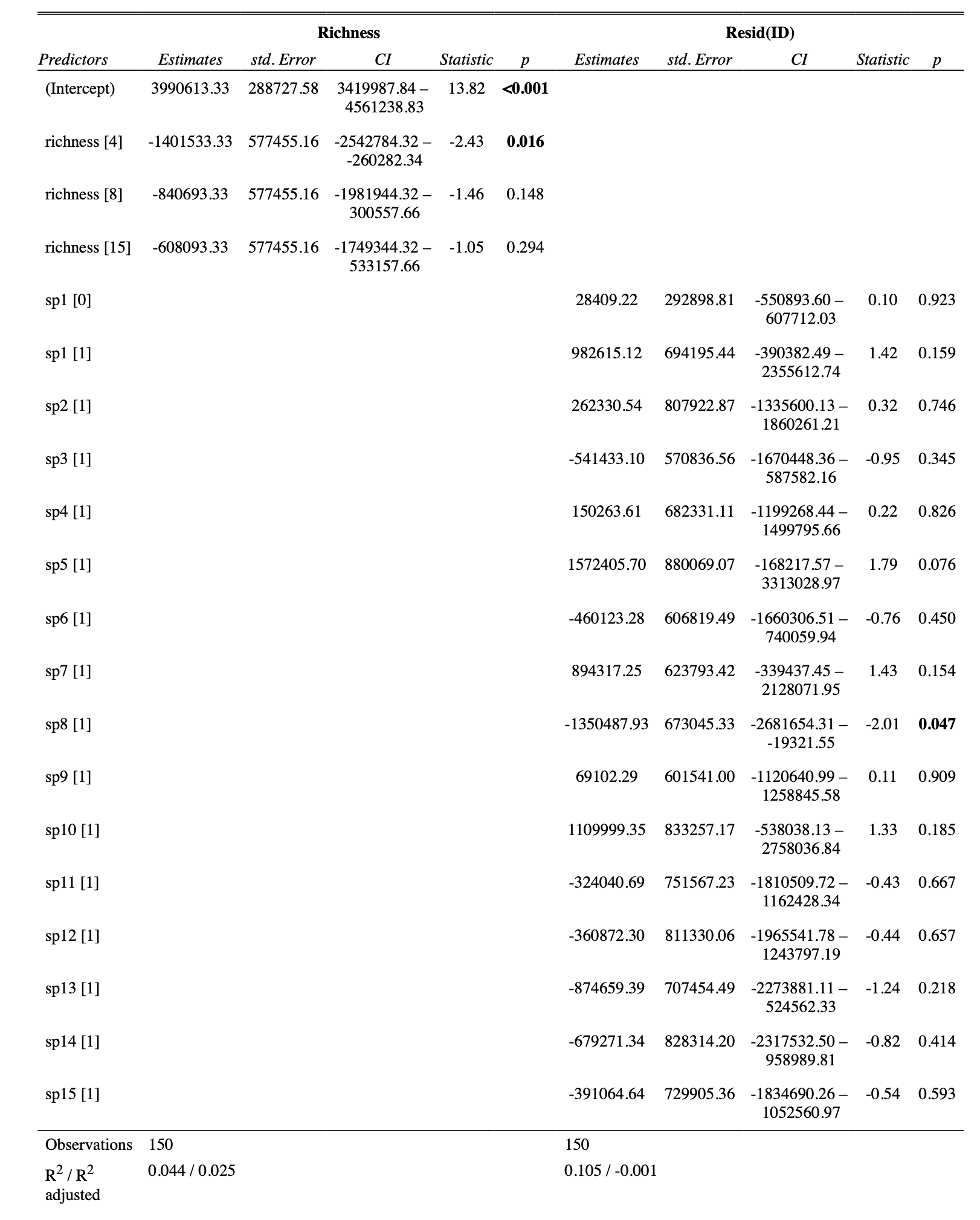


**Table S4** Tukey post-hoc test *p*-values for linear model testing the effect of community richness on producer and non-producer genotypes, grown under single genotype conditions.

| ***Comparison*** | ***p.value*** |
| --- | --- |
| **(richness2 Non-producer) - (richness4 Non-producer)** | **0.030801** |
| (richness2 Non-producer) - (richness8 Non-producer) | 0.860812 |
| (richness2 Non-producer) - (richness15 Non-producer) | 0.464574 |
| **(richness2 Non-producer) - richness2 Producer** | **2.83E-06** |
| (richness2 Non-producer) - richness4 Producer | 0.531098 |
| (richness2 Non-producer) - richness8 Producer | 0.10555 |
| (richness2 Non-producer) - richness15 Producer | 0.999999 |
| (richness4 Non-producer) - (richness8 Non-producer) | 0.747222 |
| (richness4 Non-producer) - (richness15 Non-producer) | 0.961599 |
| (richness4 Non-producer) - richness2 Producer | 0.999956 |
| (richness4 Non-producer) - richness4 Producer | 0.813516 |
| (richness4 Non-producer) - richness8 Producer | 0.999904 |
| (richness4 Non-producer) - richness15 Producer | 0.188923 |
| (richness8 Non-producer) - (richness15 Non-producer) | 0.999371 |
| (richness8 Non-producer) - richness2 Producer | 0.74805 |
| (richness8 Non-producer) - richness4 Producer | 0.999795 |
| (richness8 Non-producer) - richness8 Producer | 0.778723 |
| (richness8 Non-producer) - richness15 Producer | 0.9763 |
| (richness15 Non-producer) - richness2 Producer | 0.980427 |
| (richness15 Non-producer) - richness4 Producer | 1 |
| (richness15 Non-producer) - richness8 Producer | 0.997742 |
| (richness15 Non-producer) - richness15 Producer | 0.510399 |
| richness2 Producer - richness4 Producer | 0.965246 |
| richness2 Producer - richness8 Producer | 1 |
| richness2 Producer - richness15 Producer | 0.119548 |
| richness4 Producer - richness8 Producer | 0.995222 |
| richness4 Producer - richness15 Producer | 0.840272 |
| richness8 Producer - richness15 Producer | 0.387082 |

**Table S5** Tukey post-hoc test *p*-values for linear model testing the effect of community richness on final proportion of non-producer genotypes, where producers and non-producers are grown together (i.e. mixed genotype condition).

| ***Comparison*** | ***p.value*** |
| --- | --- |
| richness2 - richness4 | 0.238415 |
| richness2 - richness8 | 0.177196 |
| richness2 - richness15 | 0.014412 |
| richness4 - richness8 | 0.998563 |
| richness4 - richness15 | 0.644524 |
| richness8 - richness15 | 0.752729 |
